# STAT3 Regulates Monocyte TNF-Alpha Production in Systemic Inflammation Caused by Cardiac Surgery with Cardiopulmonary Bypass

**DOI:** 10.1371/journal.pone.0035070

**Published:** 2012-04-10

**Authors:** Petrus R. de Jong, Alvin W. L. Schadenberg, Theo van den Broek, Jeffrey M. Beekman, Femke van Wijk, Paul J. Coffer, Berent J. Prakken, Nicolaas J. G. Jansen

**Affiliations:** 1 Department of Pediatric Intensive Care, University Medical Center Utrecht, Wilhelmina Children's Hospital, Utrecht, The Netherlands; 2 Deptartment of Pediatric Immunology, Center for Molecular and Cellular Intervention, University Medical Center Utrecht, Wilhelmina Children's Hospital, Utrecht, The Netherlands; 3 Department of Immunology, University Medical Center Utrecht, Utrecht, The Netherlands; McGill University, Canada

## Abstract

**Background:**

Cardiopulmonary bypass (CPB) surgery initiates a controlled systemic inflammatory response characterized by a cytokine storm, monocytosis and transient monocyte activation. However, the responsiveness of monocytes to Toll-like receptor (TLR)-mediated activation decreases throughout the postoperative course. The purpose of this study was to identify the major signaling pathway involved in plasma-mediated inhibition of LPS-induced tumor necrosis factor (TNF)-α production by monocytes.

**Methodology/Principal Findings:**

Pediatric patients that underwent CPB-assisted surgical correction of simple congenital heart defects were enrolled (n = 38). Peripheral blood mononuclear cells (PBMC) and plasma samples were isolated at consecutive time points. Patient plasma samples were added back to monocytes obtained pre-operatively for *ex vivo* LPS stimulations and TNF-α and IL-6 production was measured by flow cytometry. LPS-induced p38 mitogen-activated protein kinase (MAPK) and nuclear factor (NF)-κB activation by patient plasma was assessed by Western blotting. A cell-permeable peptide inhibitor was used to block STAT3 signaling. We found that plasma samples obtained 4 h after surgery, regardless of pre-operative dexamethasone treatment, potently inhibited LPS-induced TNF-α but not IL-6 synthesis by monocytes. This was not associated with attenuation of p38 MAPK activation or IκB-α degradation. However, abrogation of the IL-10/STAT3 pathway restored LPS-induced TNF-α production in the presence of suppressive patient plasma.

**Conclusions/Significance:**

Our findings suggest that STAT3 signaling plays a crucial role in the downregulation of TNF-α synthesis by human monocytes in the course of systemic inflammation *in vivo*. Thus, STAT3 might be a potential molecular target for pharmacological intervention in clinical syndromes characterized by systemic inflammation.

## Introduction

Cardiopulmonary bypass-assisted surgery initiates a systemic inflammatory response induced by extrinsic (*e.g.* anesthesia, contact activation within the extracorporeal circuit, endotoxemia) and intrinsic (*e.g.* tissue damage, endothelial cell activation, ischemia-reperfusion injury of myocardium) factors [1]–[3]. Monocytes are important players in systemic inflammation and the main producers of pro- and anti-inflammatory cytokines upon activation of innate pattern recognition receptors [4]. Significant changes in surface biomarkers on circulating monocytes such as HLA-DR [5], [6] and chemokine receptor CX3CR1 [7] have been observed in critical illness. Moreover, monocytes activated by the extracorporeal circuit extravasate to peripheral tissues with upregulation of adhesion molecule CD11b [8]. During this dysregulation of inflammatory homeostasis, increased levels of pro-inflammatory plasma mediators such as TNF-α, IL-6 and IL-8 are joined by anti-inflammatory cytokines such as IL-10 and TGF-β [9]–[12]. Importantly, the net effect of these circulating inflammatory mediators appears to be biased towards inhibition of innate immune cells, thereby providing timely negative feedback. However, the molecular and cellular mechanisms responsible for suppression of the immune system after on-pump cardiac surgery remain unclear [13].

The anti-inflammatory phase in systemic inflammation is associated with a reduced TLR responsiveness of monocytes [14], [15]. Monocytes respond to LPS stimulation through the association of LPS/LPS-binding protein (LBP) with CD14 and TLR4 [16], [17], which results in NF-κB activation. Altered monocyte reactivity to LPS after on-pump cardiac surgery by plasma mediators may therefore be caused by reduced availability of TLR ligands (*i.e.* free LPS), by upregulation of circulating LBP [18] or lipoproteins [19]. Alternative explanations include downregulation of TLR4 and the resulting inhibition of downstream signaling cascades [20], [21], prevention of IκB-α degradation, the negative regulator of NF-κB [22], [23], or finally, the effects of signaling cascades [*e.g.* Signal transducer and activator of transcription (STAT)3] activated by the prototypic anti-inflammatory cytokine IL-10 [14].

In the present study, we evaluated these possibilities in order to identify the molecular mechanism behind the diminished response of monocytes to LPS stimulation during human systemic inflammation *in vivo*. Set against (pre-)clinical sepsis models, CPB-assisted cardiac surgery allows serial sampling of cells and plasma from the incitement, expansion, up to the resolution phase of human systemic inflammation, as previously shown [24]. Only patients with a favorable outcome were included in order to provide a controlled system of inflammatory evolution. We tested the capability of patient plasma isolated at different time points to inhibit LPS-induced TNF-α and IL-6 synthesis by monocytes. Subsequently, we tested the requirement of IL-10/STAT3 signaling for the effects of anti-inflammatory plasma on monocytes *ex vivo*.

## Results

### Activation of the innate immune system after on-pump cardiac surgery

**Figure 1:**
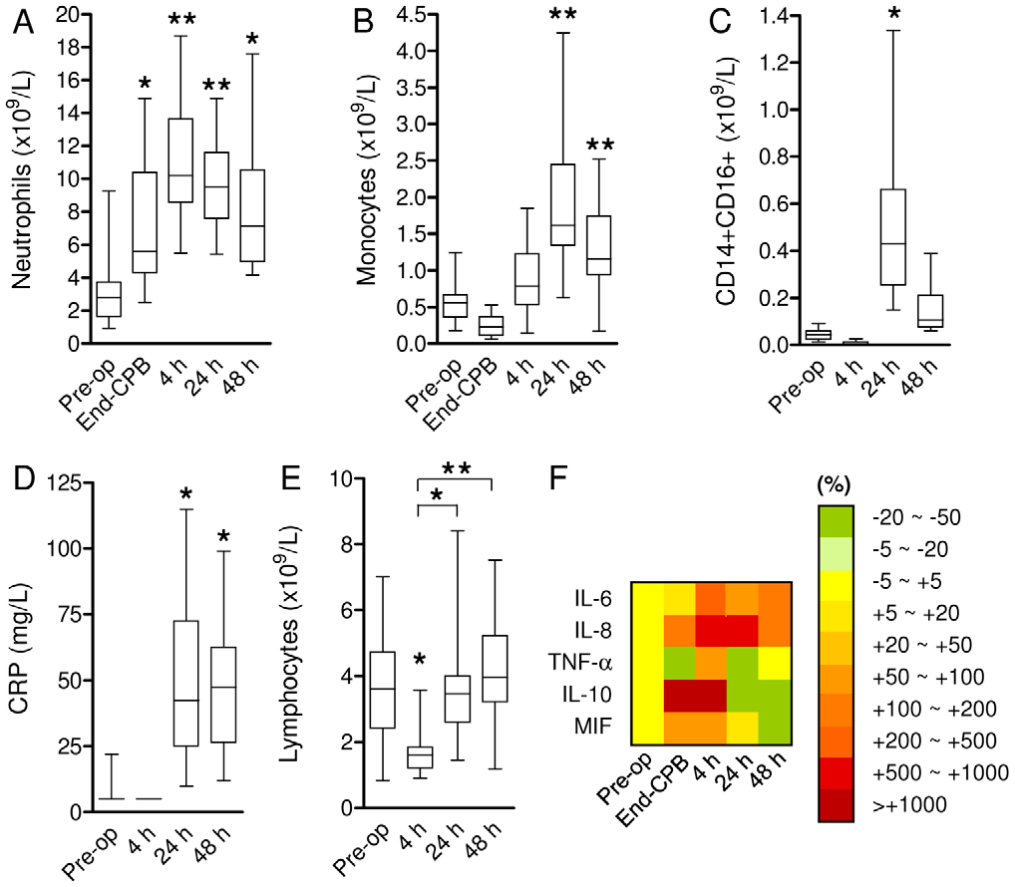
Inflammatory events induced by CPB surgery. Increased mean neutrophil (**A**) and monocyte (**B**) counts after on-pump cardiac surgery (n = 21 and n = 24, respectively). **C**. Increased numbers of circulating CD14+CD16+ monocytes after CPB surgery (n = 14). **D**. Increased mean C-reactive protein (CRP) levels in patient blood samples post-surgery (n = 22). **E**. Lymphopenia was observed 4 h post-surgery (n = 27). Box-and-whiskers plots. *P<0.01, **P<0.001 vs. pre-op (ANOVA). **F**. Cyto- and chemokine color profiles of plasma samples (n = 12) obtained at indicated time points, represented as % change compared to baseline. MIF: Macrophage migration inhibitory factor.

As expected, cardiac surgery led to *in vivo* activation of the innate immune system. Mean cell counts increased significantly 24 h after surgery for both the neutrophil (9.79±2.74 vs. 3.10±1.94·10^9^/L, **Fig. 1A**) and monocyte (1.87±0.89 vs. 0.57±0.25·10^9^/L, **Fig. 1B**) populations compared to baseline. Accordingly, the pro-inflammatory CD14+CD16+ monocyte subpopulation had expanded significantly 24 h after surgery (0.51±0.34 vs. 0.044±0.025·10^9^/L; **Fig. 1C**). These events were paralleled by elevated plasma levels of C-reactive protein 24–48 h after surgery (**Fig. 1D**), whereas we observed a transient lymphopenia 4 h after surgery (**Fig. 1E**). Analysis of plasma samples by multiplex immunoassay showed a marked increase of biomarkers that have been associated with a deleterious course in human systemic inflammation [25], including IL-6, IL-8, TNF-α, MIF (all pro-inflammatory) and IL-10 (anti-inflammatory, **Fig. 1F**). Thus, on-pump cardiac surgery leads to a temporary, controlled activation of the innate immune system with both strong pro- and anti-inflammatory signals.

### Inhibition of LPS-induced monocyte TNF-α synthesis by post-perfusion plasma

**Figure 2:**
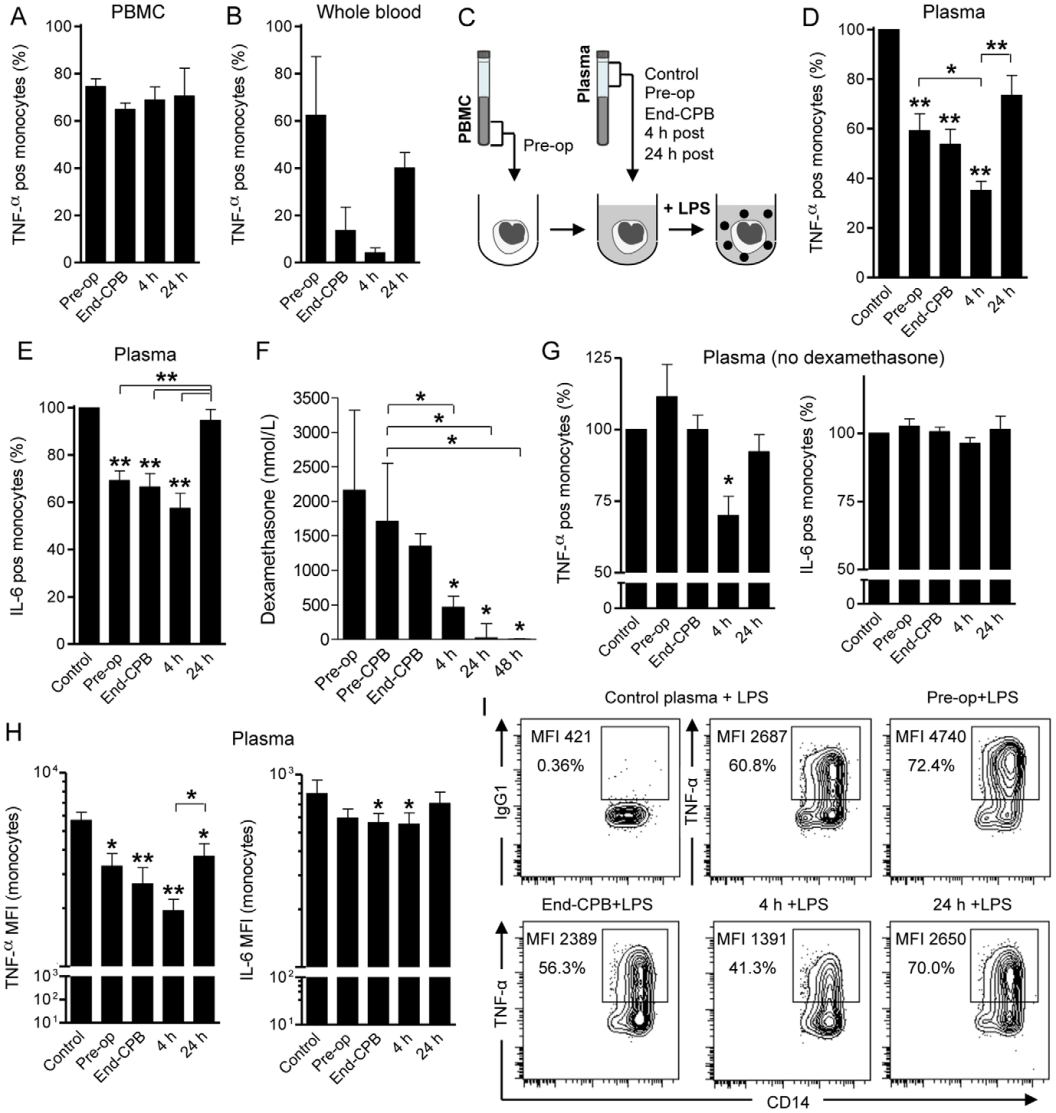
Post-perfusion plasma suppresses LPS-induced TNF-α production by monocytes. **A**. Percentage of TNF-α producing cells in the monocyte population after *ex vivo* LPS stimulation (100 ng/mL) of patient PBMC isolated at various time points (n = 4). **B**. Reduced TNF-α synthesis by monocytes after LPS (10 ng/mL) stimulation in whole blood assays with patient samples obtained at the indicated time points (n = 5). **C**. Experimental setup for experiments shown in D,E,G-I. In short, patient PBMC obtained before surgery (Pre-op) were mixed with control (pooled AB plasma from healthy donors) or autologous patient plasma samples obtained at indicated time points, followed by LPS (100 ng/mL) stimulation for 4 h. Monocyte populations (CD14/SSC gate) were then analyzed for intracellular TNF-α and IL-6 synthesis. **D**. Significantly reduced production of TNF-α by monocytes after LPS stimulation in the presence of plasma samples from different sources (n = 13). Shown are percentages of TNF-α producing monocytes relative to control (100%). *P<0.05, **P<0.001 vs. control (ANOVA). **E**. Percentages of IL-6 producing monocytes as in D. **P<0.001 vs. control (ANOVA). **F**. Dexamethasone levels in patient plasma samples as measured by radio-immunoassay (n = 9). Median ± interquartile range. *P<0.05 vs. pre-op (ANOVA). **G**. Production of TNF-α and IL-6 by monocytes after LPS stimulation in the presence of dexamethasone-free plasma samples (n = 4). *P<0.05 vs. control (ANOVA). **H**. Mean fluorescence intensities (MFI) of TNF-α and IL-6 in monocytes after LPS stimulation in different plasma milieus (n = 7). *P<0.05, **P<0.001 vs. control (ANOVA). **I**. Representative flow cytometry results (contour plots) of the LPS-induced TNF-α production by monocytes in the presence of control or patient plasma (Pre-op, End-CPB, 4 h or 24 h post-perfusion plasma from a No-dexamethasone patient). Isotype control: mouse IgG1. Data represented as mean ± SEM, unless otherwise indicated.

Next, we assessed the functional consequences of the dramatic peri- and postoperative release of inflammatory mediators on TLR-mediated monocyte activation. To study this, we stimulated thawed PBMC from patients obtained at various time points with *E. coli* LPS for 4 h in standard culture medium. Monocytes were the major responders to LPS-stimulation in PBMC as determined by intracellular TNF-α synthesis measured by FACS. However, we found only a marginal decrease in TNF-α production by patient monocytes in the course of CPB surgery (**Fig. 2A**). Accordingly, TLR4 expression levels on monocytes did not significantly change during the study period (TLR4 MFI Pre-op, End-CPB, 24 h and 48 h after surgery was 2.4±1.3, 2.3±1.1, 2.6±1.5 and 2.3±1.6, respectively). We then stimulated fresh whole blood samples obtained from patients at consecutive time-points with LPS *ex vivo*. Importantly, we now found a marked decrease of monocyte TNF-α production, which was maximal 4 h after surgery compared to baseline (**Fig. 2B**). These findings suggested that, although the intrinsic capacity of monocytes to respond to LPS did not change, plasma factors released in the course of on-pump cardiac surgery might influence their capacity to synthesize TNF-α.

To test this, we next stimulated thawed patient PBMC isolated before surgery with LPS in the presence of autologous plasma obtained at different time points or with plasma from healthy donors (control). Importantly, by using the same monocyte population for all experimental conditions (see experimental setup in **Fig. 2C**), we could specifically address the regulatory role of plasma components released in the course of human systemic inflammation on monocytes. As shown in **Fig. 2D**, we found significantly reduced TNF-α production in the presence of plasma obtained before surgery, at the end of CPB and maximal suppression mediated by 4 h post-surgery plasma (all *P*<0.001 vs. control). Importantly, the number of TNF-α positive LPS-stimulated monocytes in the presence of 4 h post-surgery plasma was significantly lower compared to pre-operative and 24 h post-surgery plasma (*P*<0.05 and *P*<0.001, respectively). Surprisingly, we did not observe a similar inhibitory effect of 4 h post-surgery plasma on IL-6 synthesis (**Fig. 2E**). Analysis of the mean fluorescence intensities of TNF-α and IL-6 in LPS-stimulated monocytes to compare their respective expression levels in the different plasma milieus reproduced the same results (**Fig. 2H**). Thus, plasma mediators released in the circulation 4 h after open heart surgery strongly suppressed LPS-induced TNF-α but not IL-6 synthesis by monocytes.

Since all patients analyzed had received dexamethasone pre-operatively, we had to exclude that this anti-inflammatory agent influenced our *ex vivo* monocyte assays. We therefore first measured dexamethasone levels in consecutive patient plasma samples and found that these were maximal in pre-operative samples, but already significantly reduced 4 h post-surgery (**Fig. 2F**). To further exclude the potential influence of steroids on the effects of 4 h post-perfusion plasma, we enrolled a control group that did not receive dexamethasone before surgery. The clinical characteristics of these patients were comparable to the previously analyzed cohort of patients (**Table 1**). We repeated the *ex vivo* plasma assays as before and analyzed LPS-induced TNF-α and IL-6 production by monocytes. Again, we found a significant effect of 4 h post-perfusion plasma samples on TNF-α production by monocytes (**Fig. 2G**). However, the inhibitory effects of Pre-op and End-CPB plasma samples on TNF-α synthesis were not found in the absence of dexamethasone. Moreover, there was no suppression of IL-6 in any of the steroid-free conditions tested (**Fig. 2G**). Representative results of a patient from the No-dexamethasone group are shown in **Fig. 2I**. We inferred from these data that 4 h post-perfusion plasma has a unique inhibitory effect on LPS-induced TNF-α but not IL-6 synthesis by monocytes.

**Table 1:** Patient characteristics.

	Dexamethasone	No-Dexamethasone	P-value
Age (mo)	12±34	7±6	0.14
Male / female	19/15	1/3	
VSD	16	3	
ASD	12	1	
AVSD	2		
Aortic valvuloplasty	2		
Extracardiac conduit	1		
CoA	1		
Duration of CPB (min)	52±28	49±29	0.82
Duration of ACC (min)	32±20	42±17	0.26
PICU stay (days)	2±1.2	1±0	0.17

Age, CPB, ACC and PICU durations represented as median ± SD. ACC: aortic crossclamping, ASD: atrial septum defect, AVSD: atrioventricular septum defect, CoA: Coarctation aorta, CPB: cardiopulmonary bypass, Extracardiac conduit change due to stenosis after Fontan procedure, PICU: pediatric intensive care unit, VSD: ventricular septum defect. No significant differences were found between both patient groups (Mann-Whitney test).

### Normal activation of p38 MAPK and NF-κB in the presence of post-perfusion plasma

**Figure 3:**
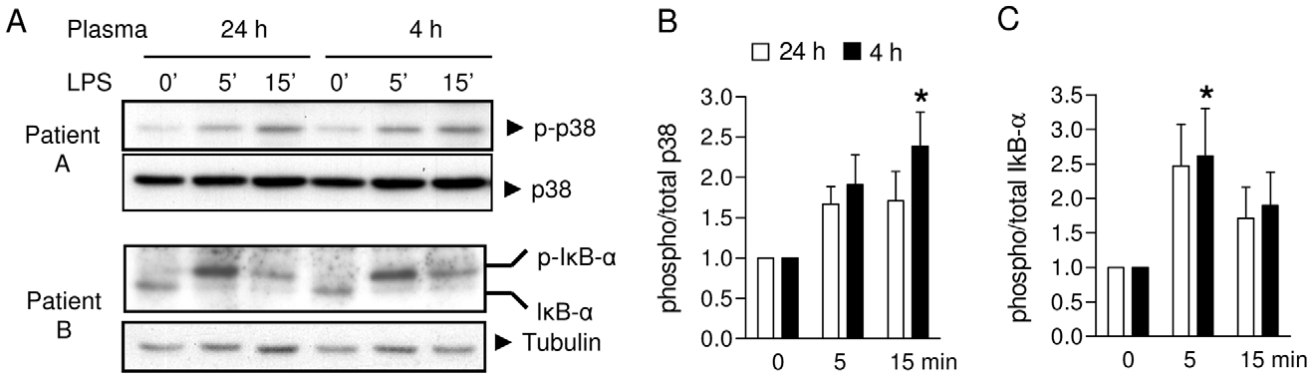
Post-perfusion plasma does not interfere with p38 MAPK or NF-κB activation. Representative examples (**A**) and densitometric analyses (**B–C**) of LPS-induced p38 MAPK and IκB-α phosphorylation in monocytes in the presence of 24 h (control) or 4 h post-surgery plasma. Tubulin: loading control. Mean ± SEM (n = 4). *P<0.05 vs. 0 min (ANOVA).

Next, we sought to elucidate the molecular mechanisms that could account for the suppression of 4 h post-surgery plasma on LPS-induced TNF-α production by monocytes. To test whether this could be explained by either sequestration of LPS in post-surgery plasma or reduced TLR4 expression on monocytes, we evaluated for differences in activation of signaling pathways downstream of TLR4. To this end, we compared the effects of 4 h vs. 24 h post-perfusion plasma samples from the same patient, since the latter did not significantly reduce LPS-induced TNF-α synthesis (see **Fig. 2G**). All three MAPK pathways *i.e.* p38, JNK/SAPK and ERK are activated by LPS in monocytes [26]. Since we found that the p38 MAPK pathway was most potently activated by LPS, we assessed the activation of p38 MAPK in purified monocytes isolated from healthy donors stimulated with LPS in the presence of patient plasma obtained 4 h or 24 h (control) post-surgery. As shown in **Fig. 3A**, there was no attenuation of p38 activation in monocytes after LPS stimulation in the presence of 4 h post-surgery plasma compared to control plasma. In contrast, densitometric analysis of Western blots from 4 different patients showed even slightly increased p38 MAPK phosphorylation in the presence of suppressive 4 h post-surgery plasma (**Fig. 3B**). IκB-α negatively regulates NF-κB by sequestering this transcription factor in the cytosol [27]. LPS-mediated phosphorylation of IκB-α induces its ubiquitination and degradation, resulting in the release of NF-κB. Evaluation of IκB-α phosphorylation after LPS stimulation showed similar kinetics in the presence of either 4 h or 24 h post-surgery plasma (**Fig. 3A,C**). Thus, we inferred from these results that suppression of LPS-induced TNF-α production by monocytes mediated by 4 h post-surgery plasma is not due to reduced TLR4 and subsequent p38 MAPK and NF-κB activation.

### A regulatory role of STAT3 signaling induced by inhibitory post-perfusion plasma

**Figure 4:**
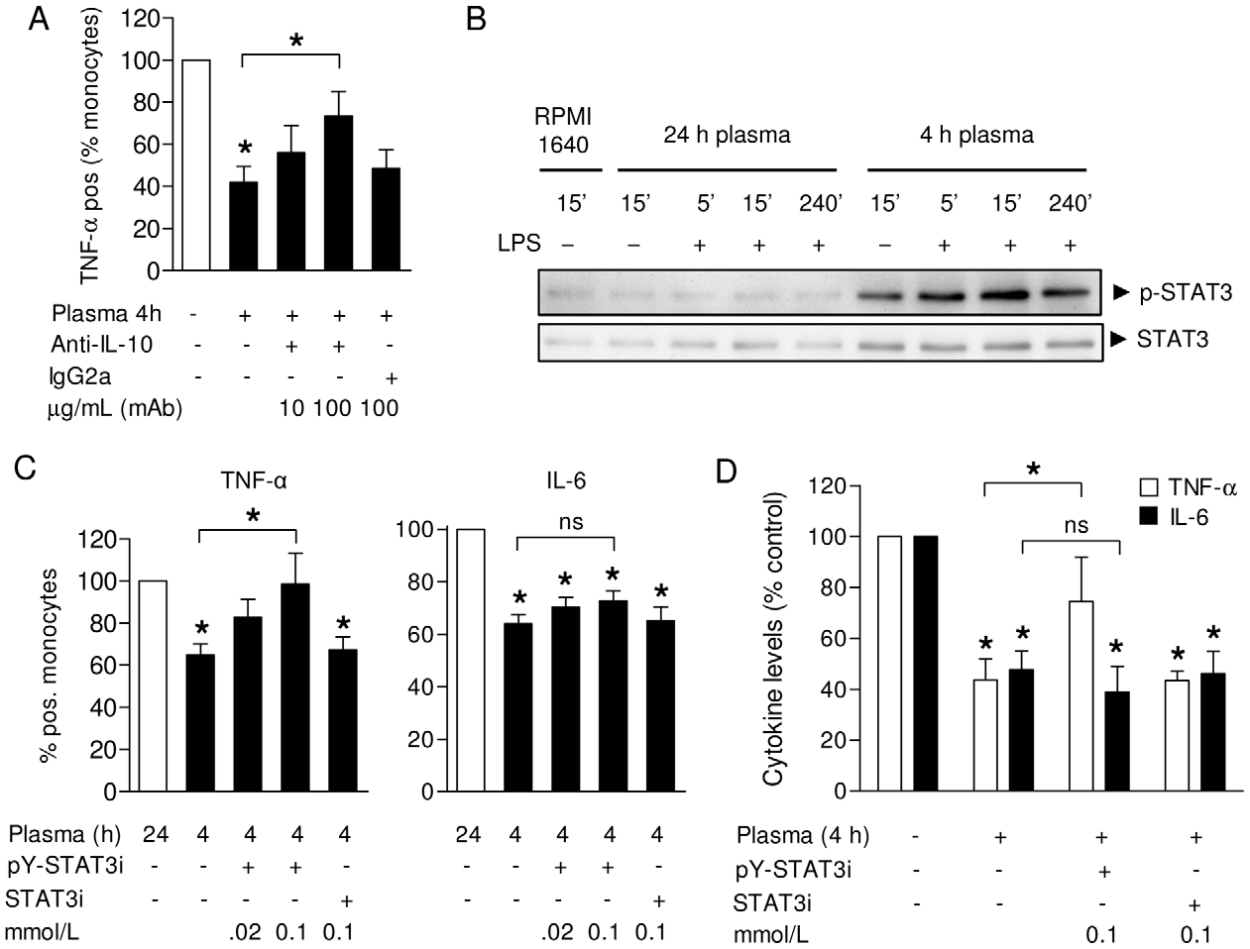
STAT3 signaling is required for the suppressive effects of post-perfusion plasma on TNF-α production. **A**. Pre-treatment of 4 h post-surgery plasma samples with anti-IL-10 partially restored TNF-α production by patient monocytes in response to LPS (n = 10). Control: plasma from healthy donors. **B**. Activation of STAT3 in monocytes by incubation with suppressive (4 h post-perfusion) but not control (24 h post-perfusion) plasma. Cells were incubated in the absence or presence of LPS to match the experimental setup as in Fig. 2. **C**. Pre-treatment of patient PBMC with active STAT3 inhibitor (pY-STAT3i) but not control peptide (STAT3i) before LPS stimulation in the presence of post-surgery plasma restored TNF-α synthesis (left panel), in contrast to IL-6 (right panel). Shown are percentages of TNF-α and IL-6 producing monocytes normalized to control (24 h post-surgery) plasma (n = 8). **D**. TNF-α and IL-6 levels measured in supernatants of LPS-stimulated mononuclear cells after pre-treatment with STAT3 inhibitor or control peptide, in the presence of 4 h post-surgery plasma (n = 8). Cytokine levels were normalized to LPS stimulation in control plasma from healthy donors due to interassay variability. All results are depicted as mean ± SEM. *P<0.05 vs. control condition (ANOVA), ns: not significant.

We next set out to assess the role of immunomodulatory cytokines in our system. As shown above, we identified high levels of the anti-inflammatory cytokine IL-10 in these plasma samples (**Fig. 1F**). As monocytes/macrophages have been shown to be both the main producers [28] and target cells of IL-10 [29], we first evaluated the effect of IL-10 neutralization. Plasma samples obtained 4 h post-surgery were pre-treated with anti-hIL-10 mAb (10 or 100 µg/mL), or the appropriate isotype control (IgG2a, 100 µg/mL), before adding these samples back to PBMC in the presence of LPS. As shown in **Fig. 4A**, we found that neutralization of IL-10 partially reversed the inhibitory effects of 4 h post-surgery plasma on TNF-α synthesis by monocytes.

IL-10 activates the JAK1/STAT3 pathway by signaling through the IL-10 receptor (IL-10R) in mononuclear cells [30], [31]. This IL-10R/STAT3 signaling axis results in the upregulation of various anti-inflammatory proteins that can inhibit pro-inflammatory cytokine synthesis [32], [33]. Indeed, we found activation of STAT3 in monocytes by incubation with plasma isolated 4 h but not 24 h post-perfusion regardless of the presence of LPS (representative example in **Fig. 4B**). Therefore, we next assessed the functional role of STAT3 signaling in monocytes with regard to the suppressive effects of post-perfusion plasma on cytokine production. We pre-treated patient PBMC with a cell-permeable STAT3 inhibitor peptide (phosphorylated peptide, pY-STAT3i) that contains a membrane translocating sequence that prevents nuclear translocation of STAT3 dimers [34]. After pre-treatment with pY-STAT3i or non-phosphorylated control peptide (STAT3i), the cells were again stimulated with LPS in the presence of 4 h post-perfusion plasma and the results were compared to those obtained with 24 h post-surgery (control) plasma. We found that STAT3 inhibition restored TNF-α production in the presence of suppressive patient plasma (**Fig. 4C** **, left panel**), but did not affect IL-6 synthesis (**Fig. 4C** **, right panel**). STAT3 inhibition also restored levels of TNF-α, but not IL-6, in supernatants of LPS-stimulated mononuclear cells incubated in the presence of 4 h post-perfusion plasma (**Fig. 4D**). In all experiments, pre-treatment with control peptide had no effect on cytokine production (**Fig. 4C,D**). Taken together, our findings suggest that STAT3 mediates the suppressive effects of plasma mediators (released shortly after CPB surgery) on TNF-α, but not IL-6, synthesis by monocytes.

## Discussion

A suppressed immune system after cardiac surgery is extensively described in both adult and pediatric patients and is associated with an enhanced risk of nosocomial infections and prolonged hospital stay [35], [36]. Previous studies identified both phenotypic cellular changes, such as HLA-DR expressed on monocytes [5] and soluble factors including IL-10 [14], [37], to be associated with clinical outcome. Our data showed a transient suppression of monocyte function in the circulation after open heart surgery, which was mainly caused by plasma components (**Fig. 2**). Previous dissections of signaling cascades responsible for suppression of the innate immune system in systemic inflammation have lead to the concept of ‘endotoxin tolerance’, particularly in human endotoxemia and sepsis. These conditions are associated with the upregulation of intracellular negative regulators of TLR4 signaling, including IL-1R-associated kinase (IRAK)-M [38], MyD88s and single immunoglobulin interleukin-1 receptor-related molecule (SIGIRR) [39]. However, we found that major signaling pathways downstream of TLR4 (*i.e.* p38 MAPK and NF-κB activation) were unimpaired in the presence of suppressive patient plasma (**Fig. 3**). This suggests that the suppression of LPS-induced TNF-α production by monocytes in our model was not explained by endotoxin tolerance.

Transcriptional activity of STAT3 in macrophages and neutrophils has been shown to be essential for the orchestration of anti-inflammatory responses in experimental models of systemic inflammation [40], [41]. Currently, there is limited information on the role of STAT3 in anti-inflammatory feedback on innate immune cells in human (sterile) systemic inflammation [42], [43]. Here we demonstrate a crucial role for STAT3 in the suppression of TNF-α synthesis by monocytes in the course of systemic inflammation associated with on-pump cardiac surgery in a well described pediatric patient population (**Fig. 4**). JAK1/STAT3 signaling has been studied broadly in primary mononuclear cells *in vitro* and both JAK1 and STAT3 are required for IL-10 mediated inhibition of LPS-induced TNF-α production [44]. On-pump cardiac surgery has been shown to induce the release of cytokines (IL-6, IL-10) associated with JAK1/STAT3 signaling [10], [14], [45], as confirmed in the present study (**Fig. 1F**). Neutralization of IL-10 in suppressive plasma samples partially reversed its inhibitory effects on LPS-induced TNF-α synthesis (**Fig. 4A**), which suggested involvement of downstream JAK1/STAT3 signaling. We subsequently found that pre-treatment of monocytes with a specific STAT3 peptide inhibitor *ex vivo* indeed restored TNF-α (but not IL-6) synthesis by monocytes (**Fig. 4C,D**). STAT3 is a critical signaling hub used by both pro- and anti-inflammatory signals mediated by IL-6 and IL-10, respectively [46], [47]. These apparent paradoxical inputs are differentially regulated by suppressor of cytokine signaling (SOCS)3 [32]. While the IL-6 receptor is susceptible to feedback inhibition by SOCS3, the IL-10R is not. The IL-10R-induced STAT3 pathway induces a transcriptional program of anti-inflammatory gene products resulting in the repression of pro-inflammatory transcripts [48], [49]. Surprisingly, STAT3 inhibition in our study failed to reverse the suppression of IL-6 production by monocytes in the presence of post-perfusion plasma, in contrast to TNF-α (**Fig. 4C,D**). This unexpected finding warrants further dissection of the exact molecular mechanisms used by STAT3 to selectively regulate TNF-α synthesis in human monocytes. Besides an important regulator of inflammation, STAT3 plays a potential role in cytoprotection and regeneration. With regard to cardiac surgery, STAT3 contributes to cardioprotective mechanisms in ischemia-reperfusion injury [50], [51], with a major role for IL-6 in the induction of this pathway [52]. Thus, our results add another feature to the multifaceted properties of STAT3 signaling in different cell types to promote tissue homeostasis after cardiac surgery.

Pharmacological agents administered during and after the surgical and anesthesiological procedures could have affected our *ex vivo* plasma assays. In particular, the pre-operative administration of dexamethasone (standard practice for this type of surgery in our hospital and most other institutions [53]) may be of influence, as corticosteroids are known for their potent anti-inflammatory effects on innate immune cells. However, we found that the circulating levels of dexamethasone were already significantly reduced 4 h after surgery (**Fig. 2F**). More importantly, we repeated the key experiments with plasma samples obtained from patients that did not receive steroids before the procedure (clinical characteristics in **Table 1**). Steroid-free plasma isolated shortly (4 h) after open heart surgery was still able to suppress LPS-induced TNF-α production by monocytes (**Fig. 2G**). These results also suggest that the suppressive plasma components were not indirectly induced by steroids. By comparing the results obtained with plasma with and without dexamethasone (compare **Fig. 2D, E** and **Fig. 2G**, respectively), we inferred that only the suppressive effect of 4 h post-perfusion plasma on TNF-α synthesis was likely caused by endogenous plasma factors. No effect on IL-6 synthesis by monocytes was found with steroid-free plasma which is consistent with our observations that abrogation of IL10/STAT3 signaling did not affect IL-6 production in monocytes (**Fig. 4**). Please note that due to limited availability of these steroid-free samples we could not perform additional experiments with IL-10 neutralizing antibodies and STAT3 inhibitor peptide.

We demonstrated a non-redundant role for STAT3 in mediating negative feedback on LPS-induced monocyte TNF-α (but not IL-6) production after on-pump cardiac surgery. This supports the concept of specific monocyte reprogramming in the course of human systemic inflammation, rather than general immune suppression. Our findings suggest that functional modulation of STAT3 activity offers a potential target for molecular intervention in suppressed states of the innate immune system in human disease.

## Materials and Methods

### Ethics Statement

Written informed consent was obtained from the parents of children participating in the study. A medical ethics committee (Medische Ethische Toetsings Commissie UMC Utrecht) approved this study (METC 03/049-K, 04/144-K UMC Utrecht, The Netherlands) and all procedures were in accordance with institutional guidelines.

### Study population, surgical and anesthesiological procedures

Children admitted to our hospital for surgical repair of relatively simple congenital heart defects with an expected rapid recovery were enrolled. For this purpose, we only included patients who underwent a surgical procedure from RACHS-1 (Risk Adjustment for Congenital Heart Surgery) score of 2 or less [54]. Patients that had signs of infection or a documented immunodeficiency were excluded. A total of 38 children were enrolled in the study and all experienced an uneventful peri- and postoperative clinical course. Detailed clinical characteristics are depicted in Table 1. The surgical, anesthesiological and cardiopulmonary bypass procedures have been published previously [24]. Briefly, general anesthesia was always implemented using a standard technique involving sufentanil, midazolam, pancuronium, dopamine and milrinone. All patients received 48 hours perioperative antibiotic prophylaxis with Cefazolin. Patients receiving dexamethasone were given a single dose of dexamethasone (1 mg/kg) after induction of anesthesia. Four patients received no steroids before the procedure. Non-pulsatile cardiopulmonary bypass was used, the standard pump flow rate was 2.8 liter/m^2^/min. Combined alpha and pH stat management of acid-base status was used during cardiopulmonary bypass. The cardioplegia procedure was standardized using St. Thomas' solution. After weaning from cardiopulmonary bypass all patients remained intubated and ventilated and were admitted to the pediatric intensive care for further management. At the pediatric intensive care patients were treated with milrinone, midazolam and morphine for maximally 24 hours. All patients were treated by the same surgical and anesthetic team.

### Blood sampling and cell isolation

Blood samples were obtained at the following time points: immediately after insertion of a central venous catheter during anesthetic induction (Pre-op), at the end of cardiopulmonary bypass (End-CPB), 4 h, 24 h and 48 h after surgery. At these time points, total leucocyte, neutrophil, monocyte, lymphocyte counts and C-reactive protein (CRP) levels were determined. Fresh heparinized blood samples were used for full blood assays. For all other purposes, plasma samples were prepared by centrifugation and stored at −80°C, whereas PBMC were separated by density gradient centrifugation over Ficoll-Hypaque (Amersham Pharmacia Biotech) and stored in liquid nitrogen, as previously described [55]. In some assays, pooled human AB plasma from healthy volunteers (Sanquin Blood Bank, Utrecht, The Netherlands) was used as control plasma.

### Antibodies

Fluorescently labeled or unconjugated monoclonal antibodies (mAb) directed against human CD14 (murine, clone MOP9, StemCell Technologies), mouse anti-FcγRIII/CD16 (3G8, BD Biosciences), mouse anti-CD284/TLR4 (HTA125, eBioscience), mouse anti-TNF-α (Mab11, eBioscience) and rat anti-IL-6 (MQ2-6A3, BD) were used for flow cytometry. MAbs directed against hIL-10 (JES3-19F1, rat IgG2a, BD) and rat IgG2a isotype (BD) were used for neutralization experiments. Antibodies directed against p-p38, p38, p-IκB-alpha and p-STAT3 (Cell Signaling), IκB-alpha and STAT3 (Santa Cruz) and Tubulin (Sigma) were used for Western blotting.

### Cellular assays

Whole blood stimulation assays were performed in RPMI-1640 at 1∶5 dilution. Cells were incubated with or without LPS (*Escherichia coli* O127:B8E, L4517, Sigma) at 10 ng/mL in a 96-well plate (Costar) for 4 h at 37°C, 5% CO_2_ with 100% relative humidity. Cells were then washed and stained for surface markers followed by lysis of red blood cells (BD Lysing Solution) and intracellular cytokine staining. For *ex vivo* LPS stimulation assays, PBMC from various time points were plated in a 96-well plate at 2×10^6^ cells/mL in RPMI-1640 supplemented with 2 mmol/L glutamine, 100 U/mL penicillin/streptomycin (Gibco BRL, Invitrogen) and 10% (v/v) heat-inactivated human AB serum. LPS was added (100 ng/mL LPS) for 4 h, followed by intracellular cytokine staining. For plasma assays, patient PBMC isolated before surgery were adjusted to 2×10^6^ cells/mL in supplemented RPMI-1640 (no serum). Pooled human AB plasma (control) and autologous patient plasma samples obtained at serial time-points were thawed and spun (300 g, 10 min) and the supernatants were filtered (50 µm). Plasma samples mixed with LPS (100 ng/mL end concentration) were added to equal volumes of cell suspensions (50% v/v) and incubated for 4 h at 37°C, followed by intracellular cytokine staining. For IL-10 neutralization assays, patient plasma samples were pre-incubated with anti-hIL-10 mAb (10–100 µg/mL) or IgG2a isotype (100 µg/mL) for 1 h at 4°C on a shaker. For STAT3 inhibition assays, PBMC obtained before surgery were pre-treated with 0.02 or 0.1 mM cell-permeable STAT3 Inhibitor Peptide (PpYLKTK-mts, Calbiochem) or 0.1 mM inactive control peptide (Ac-PpYLKTK-OH) for 1 h at 37°C in culture medium with 10% AB plasma. PBMC were then washed and mixed with plasma samples (4 h or 24 h post-surgery) and LPS (100 ng/mL) for a 4 h incubation period followed by intracellular cytokine staining.

### Flow cytometry

Golgistop (2 µM, BD) was added during *ex vivo* incubations with LPS. Cells were then washed, blocked with normal mouse serum followed by extracellular staining, fixation in Cytofix/Cytoperm and washing in Perm/Wash solution (Cytofix/perm kit, BD). Finally, cells were incubated with mAbs for intracellular cytokine staining, as published [24].

### Multiplex immunoassay

Multiplex immunoassay with the Bio-Plex suspension array system (Bio-Rad Laboratories) was used to measure levels of TNF-α, IL-6, IL-8, IL-10 and MIF in patient plasma samples and culture supernatants, as previously described [56].

### Dexamethasone measurements

Dexamethasone in serum was measured after diethylether extraction using an in house competitive radio-immunoassay (RIA) employing a polyclonal anti-dexamethasone-antibody (IgG dex1 lot 1301; IgG Corporation). [1,2,4,6,7-3H]-dexamethasone (TRK645, Amersham) was used as a tracer following chromatographic verification of its purity. The lower limit of detection was 20 pmol/L and intra-assay variation was <7%. All samples were included in one assay.

### Western blot analysis

Purified monocytes from healthy donors were serum starved for at least 2 h, washed and resuspended in supplemented RPMI-1640. A total of 5×10^5^ cells per condition were stimulated in the absence or presence of LPS (100 ng/mL) for 0, 5, 15 or 240 min at 37°C in the presence of 50% (v/v) patient plasma. After *in vitro* stimulation, cells were washed with cold PBS and lysed in reducing Laemmli sample buffer. Proteins were separated with SDS-PAGE, transferred to PVDF membranes, blocked with 5% BSA, followed by immunoprobing overnight at 4°C. Proteins were detected with HRP-conjugated secondary antibodies (Dako) and developed with Hyperfilm ECL (GE Healthcare). Densitometric analysis was performed with ImageQuant densitometric software (Molecular Dynamics).

### Statistical analysis

Basic descriptive statistics were used to describe the patient population. Multiple data sets were analyzed by one-way ANOVA, as indicated. Significance was accepted at *P<0.05 and **P<0.001.

## References

[1] Tomic V, Russwurm S, Moller E, Claus RA, Blaess M (2005). Transcriptomic and proteomic patterns of systemic inflammation in on-pump and off-pump coronary artery bypass grafting.. Circulation.

[2] Diegeler A, Doll N, Rauch T, Haberer D, Walther T (2000). Humoral immune response during coronary artery bypass grafting: A comparison of limited approach, “off-pump” technique, and conventional cardiopulmonary bypass.. Circulation.

[3] Chew MS, Brandslund I, Brix-Christensen V, Ravn HB, Hjortdal VE (2001). Tissue injury and the inflammatory response to pediatric cardiac surgery with cardiopulmonary bypass: A descriptive study.. Anesthesiology.

[4] Xing L, Remick DG (2003). Relative cytokine and cytokine inhibitor production by mononuclear cells and neutrophils.. Shock.

[5] Allen ML, Peters MJ, Goldman A, Elliott M, James I (2002). Early postoperative monocyte deactivation predicts systemic inflammation and prolonged stay in pediatric cardiac intensive care.. Crit Care Med.

[6] Peters M, Petros A, Dixon G, Inwald D, Klein N (1999). Acquired immunoparalysis in paediatric intensive care: Prospective observational study.. BMJ.

[7] Pachot A, Cazalis MA, Venet F, Turrel F, Faudot C (2008). Decreased expression of the fractalkine receptor CX3CR1 on circulating monocytes as new feature of sepsis-induced immunosuppression.. J Immunol.

[8] Evans BJ, Haskard DO, Finch JR, Hambleton IR, Landis RC (2008). The inflammatory effect of cardiopulmonary bypass on leukocyte extravasation in vivo.. J Thorac Cardiovasc Surg.

[9] Seghaye M, Duchateau J, Bruniaux J, Demontoux S, Bosson C (1996). Interleukin-10 release related to cardiopulmonary bypass in infants undergoing cardiac operations.. J Thorac Cardiovasc Surg.

[10] Sablotzki A, Welters I, Lehmann N, Menges T, Gorlach G (1997). Plasma levels of immunoinhibitory cytokines interleukin-10 and transforming growth factor-beta in patients undergoing coronary artery bypass grafting.. Eur J Cardiothorac Surg.

[11] Tarnok A, Schneider P (2001). Pediatric cardiac surgery with cardiopulmonary bypass: Pathways contributing to transient systemic immune suppression.. Shock.

[12] Franke A, Lante W, Fackeldey V, Becker HP, Thode C (2002). Proinflammatory and antiinflammatory cytokines after cardiac operation: Different cellular sources at different times.. Ann Thorac Surg.

[13] Wilhelm W, Grundmann U, Rensing H, Werth M, Langemeyer J (2002). Monocyte deactivation in severe human sepsis or following cardiopulmonary bypass.. Shock.

[14] Dehoux MS, Hernot S, Asehnoune K, Boutten A, Paquin S (2000). Cardiopulmonary bypass decreases cytokine production in lipopolysaccharide-stimulated whole blood cells: Roles of interleukin-10 and the extracorporeal circuit.. Crit Care Med.

[15] Borgermann J, Friedrich I, Flohe S, Spillner J, Majetschak M (2002). Tumor necrosis factor-alpha production in whole blood after cardiopulmonary bypass: Downregulation caused by circulating cytokine-inhibitory activities.. J Thorac Cardiovasc Surg.

[16] Wright SD, Ramos RA, Tobias PS, Ulevitch RJ, Mathison JC (1990). CD14, a receptor for complexes of lipopolysaccharide (LPS) and LPS binding protein.. Science.

[17] Haziot A, Tsuberi BZ, Goyert SM (1993). Neutrophil CD14: Biochemical properties and role in the secretion of tumor necrosis factor-alpha in response to lipopolysaccharide.. J Immunol.

[18] Lequier LL, Nikaidoh H, Leonard SR, Bokovoy JL, White ML (2000). Preoperative and postoperative endotoxemia in children with congenital heart disease.. Chest.

[19] Kitchens RL, Thompson PA, O'Keefe GE, Munford RS (2000). Plasma constituents regulate LPS binding to, and release from, the monocyte cell surface.. J Endotoxin Res.

[20] Dybdahl B, Wahba A, Lien E, Flo TH, Waage A (2002). Inflammatory response after open heart surgery: Release of heat-shock protein 70 and signaling through toll-like receptor-4.. Circulation.

[21] Hadley JS, Wang JE, Michaels LC, Dempsey CM, Foster SJ (2007). Alterations in inflammatory capacity and TLR expression on monocytes and neutrophils after cardiopulmonary bypass.. Shock.

[22] Shames BD, Selzman CH, Meldrum DR, Pulido EJ, Barton HA (1998). Interleukin-10 stabilizes inhibitory kappaB-alpha in human monocytes.. Shock.

[23] Takezako N, Hayakawa M, Hayakawa H, Aoki S, Yanagisawa K (2006). ST2 suppresses IL-6 production via the inhibition of IkappaB degradation induced by the LPS signal in THP-1 cells.. Biochem Biophys Res Commun.

[24] Schadenberg AWL, Vastert SJ, Evens FCM, Kuis W, van Vught AJ (2011). FOXP3+CD4+ Tregs lose suppressive potential but remain anergic during transient inflammation in human.. Eur J Immunol.

[25] Pierrakos C, Vincent JL (2010). Sepsis biomarkers: A review.. Crit Care.

[26] Guha M, Mackman N (2001). LPS induction of gene expression in human monocytes.. Cell Signal.

[27] Piao W, Song C, Chen H, Diaz MA, Wahl LM (2009). Endotoxin tolerance dysregulates MyD88- and Toll/IL-1R domain-containing adapter inducing IFN-beta-dependent pathways and increases expression of negative regulators of TLR signaling.. J Leukoc Biol.

[28] Bazzoni F, Tamassia N, Rossato M, Cassatella MA (2010). Understanding the molecular mechanisms of the multifaceted IL-10-mediated anti-inflammatory response: Lessons from neutrophils.. Eur J Immunol.

[29] Pils MC, Pisano F, Fasnacht N, Heinrich JM, Groebe L (2010). Monocytes/macrophages and/or neutrophils are the target of IL-10 in the LPS endotoxemia model.. Eur J Immunol.

[30] Williams LM, Sarma U, Willets K, Smallie T, Brennan F (2007). Expression of constitutively active STAT3 can replicate the cytokine-suppressive activity of interleukin-10 in human primary macrophages.. J Biol Chem.

[31] Williams L, Bradley L, Smith A, Foxwell B (2004). Signal transducer and activator of transcription 3 is the dominant mediator of the anti-inflammatory effects of IL-10 in human macrophages.. J Immunol.

[32] Berlato C, Cassatella MA, Kinjyo I, Gatto L, Yoshimura A (2002). Involvement of suppressor of cytokine signaling-3 as a mediator of the inhibitory effects of IL-10 on lipopolysaccharide-induced macrophage activation.. J Immunol.

[33] Lee TS, Chau LY (2002). Heme oxygenase-1 mediates the anti-inflammatory effect of interleukin-10 in mice.. Nat Med.

[34] Turkson J, Ryan D, Kim JS, Zhang Y, Chen Z (2001). Phosphotyrosyl peptides block Stat3-mediated DNA binding activity, gene regulation, and cell transformation.. J Biol Chem.

[35] Sarvikivi E, Lyytikainen O, Nieminen H, Sairanen H, Saxen H (2008). Nosocomial infections after pediatric cardiac surgery.. Am J Infect Control.

[36] Michalopoulos A, Geroulanos S, Rosmarakis ES, Falagas ME (2006). Frequency, characteristics, and predictors of microbiologically documented nosocomial infections after cardiac surgery.. Eur J Cardiothorac Surg.

[37] Allen ML, Hoschtitzky JA, Peters MJ, Elliott M, Goldman A (2006). Interleukin-10 and its role in clinical immunoparalysis following pediatric cardiac surgery.. Crit Care Med.

[38] van 't Veer C, van den Pangaart PS, van Zoelen MA, de Kruif M, Birjmohun RS (2007). Induction of IRAK-M is associated with lipopolysaccharide tolerance in a human endotoxemia model.. J Immunol.

[39] Adib-Conquy M, Adrie C, Fitting C, Gattolliat O, Beyaert R (2006). Up-regulation of MyD88s and SIGIRR, molecules inhibiting toll-like receptor signaling, in monocytes from septic patients.. Crit Care Med.

[40] Matsukawa A, Takeda K, Kudo S, Maeda T, Kagayama M (2003). Aberrant inflammation and lethality to septic peritonitis in mice lacking STAT3 in macrophages and neutrophils.. J Immunol.

[41] Takeda K, Clausen BE, Kaisho T, Tsujimura T, Terada N (1999). Enhanced Th1 activity and development of chronic enterocolitis in mice devoid of Stat3 in macrophages and neutrophils.. Immunity.

[42] Tamassia N, Calzetti F, Menestrina N, Rossato M, Bazzoni F (2008). Circulating neutrophils of septic patients constitutively express IL-10R1 and are promptly responsive to IL-10.. Int Immunol.

[43] Oiva J, Mustonen H, Kylanpaa ML, Kyhala L, Alanara T (2010). Patients with acute pancreatitis complicated by organ failure show highly aberrant monocyte signaling profiles assessed by phospho-specific flow cytometry.. Crit Care Med.

[44] Riley JK, Takeda K, Akira S, Schreiber RD (1999). Interleukin-10 receptor signaling through the JAK-STAT pathway. requirement for two distinct receptor-derived signals for anti-inflammatory action.. J Biol Chem.

[45] Ogata M, Okamoto K, Kohriyama K, Kawasaki T, Itoh H (2000). Role of interleukin-10 on hyporesponsiveness of endotoxin during surgery.. Crit Care Med.

[46] Niemand C, Nimmesgern A, Haan S, Fischer P, Schaper F (2003). Activation of STAT3 by IL-6 and IL-10 in primary human macrophages is differentially modulated by suppressor of cytokine signaling 3.. J Immunol.

[47] Yasukawa H, Ohishi M, Mori H, Murakami M, Chinen T (2003). IL-6 induces an anti-inflammatory response in the absence of SOCS3 in macrophages.. Nat Immunol.

[48] Murray PJ (2005). The primary mechanism of the IL-10-regulated antiinflammatory response is to selectively inhibit transcription.. Proc Natl Acad Sci U S A.

[49] Murray PJ (2007). The JAK-STAT signaling pathway: Input and output integration.. J Immunol.

[50] Bolli R, Stein AB, Guo Y, Wang OL, Rokosh G (2011). A murine model of inducible, cardiac-specific deletion of STAT3: Its use to determine the role of STAT3 in the upregulation of cardioprotective proteins by ischemic preconditioning.. J Mol Cell Cardiol.

[51] Heusch G, Musiolik J, Gedik N, Skyschally A (2011). Mitochondrial STAT3 activation and cardioprotection by ischemic postconditioning in pigs with regional myocardial ischemia/reperfusion.. Circ Res.

[52] Boengler K, Hilfiker-Kleiner D, Drexler H, Heusch G, Schulz R (2008). The myocardial JAK/STAT pathway: From protection to failure.. Pharmacol Ther.

[53] Checchia PA, Bronicki RA, Costello JM, Nelson DP (2005). Steroid use before pediatric cardiac operations using cardiopulmonary bypass: An international survey of 36 centers.. Pediatr Crit Care Med.

[54] Jenkins KJ, Gauvreau K, Newburger JW, Spray TL, Moller JH (2002). Consensus-based method for risk adjustment for surgery for congenital heart disease.. J Thorac Cardiovasc Surg.

[55] de Jong H, Lafeber FF, de Jager W, Haverkamp MH, Kuis W (2009). Pan-DR-binding Hsp60 self epitopes induce an interleukin-10-mediated immune response in rheumatoid arthritis.. Arthritis Rheum.

[56] de Jager W, Prakken BJ, Bijlsma JW, Kuis W, Rijkers GT (2005). Improved multiplex immunoassay performance in human plasma and synovial fluid following removal of interfering heterophilic antibodies.. J Immunol Methods.

